# Emotional dysregulation in women with endometriosis with cyclical and non-cyclical chronic pelvic pain

**DOI:** 10.1186/s12905-022-02066-5

**Published:** 2022-12-17

**Authors:** Dulce Carolina Rodríguez-Lozano, María del Pilar Meza-Rodríguez, Olivier Paul Cruz-Orozco, Brenda Sánchez-Ramírez, Andrea Olguin-Ortega, José Roberto Silvestri-Tomassoni, Guillermo Corona-Barsse, Luis Fernando Escobar-Ponce, Juan Mario Solis-Paredes, Benjamín Dominguez-Trejo, Ignacio Camacho-Arroyo

**Affiliations:** 1 Unidad de Investigación en Reproducción Humana, Instituto Nacional de Perinatología-Facultad de Química, Universidad Nacional Autónoma de México, 04510 Mexico City, (CD MX) Mexico; 2 Departamento de Neurociencias, Instituto Nacional de Perinatología, Av. Montes Urales # 800. Col. Lomas de Virreyes, 11000 Mexico City, CD MX Mexico; 3 Departamento de Ginecología, Instituto Nacional de Perinatología, Mexico City, Mexico; 4 Departamento de Genética y Genómica Humana, Instituto Nacional de Perinatología, Mexico City, Mexico; 5 Facultad de Psicología, Universidad Nacional Autónoma de México, Mexico City, Mexico

**Keywords:** Chronic pelvic pain, Anxiety, Depression, Endometriosis, Menstrual cycle, Emotions

## Abstract

**Background:**

Endometriosis is a pathophysiological condition characterized by glands and stroma outside the uterus in regions such as the bladder, ureter, fallopian tubes, peritoneum, ovaries, and even in extra pelvic sites. One of the main clinical problems of endometriosis is chronic pelvic pain (CPP), which considerably affects the patients’ quality of life. Patients with endometriosis may, cyclically or non-cyclically (80% of cases) experience CPP. High levels of anxiety and depression have been described in patients with endometriosis related to CPP; however, this has not been evaluated in endometriosis women with different types of CPP. Therefore, the research question of this study was whether there is a difference in the emotional dysregulation due to the type of pain experienced by women with endometriosis?

**Methods:**

This work was performed in the National Institute of Perinatology (INPer) in Mexico City from January 2019 to March 2020 and aimed to determine if there are differences in emotional dysregulation in patients with cyclical and non-cyclical CPP. 49 women from 18 to 52 years-old diagnosed with endometriosis presenting cyclical and non-cyclical CPP answered several batteries made up of Mini-Mental State Examination, Visual Analog Scale, Beck's Depression Inventory, State Trait-Anxiety Inventory, and Generalized Anxiety Inventory. Mann–Whitney U and Student’s t-test for independent samples to compare the difference between groups was used. Relative risk estimation was performed to determine the association between non-cyclical and cyclical CPP with probability of presenting emotional dysregulation.

**Results:**

We observed that patients with non-cyclical CPP exhibited higher levels of depression and anxiety (trait-state and generalized anxiety) than patients with cyclical pain, *p* < 0.05 was considered significant. No differences were observed in pain intensity, but there was a higher probability of developing emotional dysregulation (anxiety or depression) in patients with non-cyclical CPP. No differences were observed in cognitive impairment.

**Conclusions:**

Our data suggest that patients with non-cyclical (persistent) CPP present a higher emotional dysregulation than those with cyclical pain.

## Introduction

Endometriosis is a disease distinguished by a tissue similar to the lining of the uterus growing outside it causing pain and infertility [1, 2]. 50% of infertile women exhibit endometriosis worldwide. Therefore, it is considered the most common gynecological disease in women of reproductive age and in perimenopausal women [3]. In Mexico, epidemiological reviews have estimated an endometriosis incidence of 34.5% in women diagnosed with primary and secondary infertility at the National Institute of Perinatology [4].

Endometriosis symptoms are infertility, dyspareunia, heavy menstrual bleeding, chronic fatigue, fibromyalgia, migraine, and central sensitization syndrome [5–7]. However, the main clinical problem of endometriosis is chronic pelvic pain (CPP), which is defined as intermittent or constant pain in the lower abdomen or pelvis of at least six months, not occurring exclusively with menstruation or intercourse, and not associated with pregnancy [8, 9]. One of the most common causes of CPP in women is endometriosis (24–40%). Other associated conditions such as interstitial cystitis/bladder pain syndrome, chronic urinary tract infections, vulvodynia, irritable bowel syndrome, and inflammatory bowel disease may be comorbid with endometriosis [10–12].

CPP is a persistent and debilitating condition associated with high costs and morbidity. Significant costs are associated with CPP, including absences from work, increased surgeries, and heavy burden to the healthcare system [13]. CPP and infertility in women with endometriosis are associated with high levels of stress and uncertainty, reducing their quality of life and making challenging the performance of daily activities and the development of interpersonal relationships [14, 15]. Additionally, the difficulty experienced by these women from the onset of the first symptoms until diagnosis increases the probability of presenting emotional alterations. The average time between the onset of symptoms and the seeking help is from 3.7 to 5.7 years, extending up to 8 years for timely diagnosis [7, 16, 17].

The mechanisms by which CPP is generated in endometriosis have not been clearly defined. However, it occurs near endometriotic glands, and blood vessels in peritoneal endometriotic lesions innervated by sensory A delta, sensory C, cholinergic and adrenergic nerve fibers [18]. Nerve fiber densities are increased in the myometrium of women with endometriosis compared with those presented in women without this pathology [19, 20]. Although these nerve fibers may play an essential role in the mechanisms of pain generation in endometriosis, the emotional dysregulation can mediate the nociceptive experience by brain regions such as the anterior insula and the anterior cingulate cortex [21, 22].

Variable and broad symptoms and social implications of endometriosis have been considered disruptive to mental health, exhibiting high anguish, anxiety, depression, and chronic stress [23–26]. It has been described that the presence of CPP affects mental health [14], regardless of endometriosis stage or type [27], and it did not always decrease after medical treatment or surgery. Patients with endometriosis may experience CPP cyclically or non-cyclically (80% of cases) defined as non-menstrual pain [28, 29]; however, whether there is a difference in levels of anxiety and depression between these two patient groups has not been evaluated. Therefore, it is not known how different CPP affects the emotional state of women with endometriosis. This study aimed to determine if there are differences in emotional dysregulation in patients with cyclic and non-cyclic CPP.

## Methods

### Design of the study

We conducted a transversal study at the National Institute of Perinatology (INPer, Neuroscience Department, Mexico City) from January 2019 to March 2020. Approval from the Institution Ethical and Scientific Committee was obtained before the beginning of the study (reference number: INPer, 2019–1-51). Women with endometriosis were invited to participate in the study when coming to their gynecology interview at Department of Gynecology at INPer. Patients who voluntary participated in the study were requested to sign a written informed consent.

During the initial interviews at the Gynecology and Neuroscience Departments, we assessed the patients’ eligibility according to the inclusion criteria. Participants gynecological, sociodemographic, and psychological characteristics were recorded in a database.

### Participants

Patients recruited into the study were attending at the Gynecology Department in the INPer. The recruited population comprised women from 18 to 52 years old with a diagnosis of endometriosis (by laparoscopic or magnetic resonance) and CPP for at least 1 year. The medical staff carried out a complete clinical evaluation and an analysis of sociodemographic variables was done, including marital status, education level, and working status. Participants were asked to complete self-reported questionnaires used to measure cognitive impairment, the intensity of pelvic pain, general discomfort, depression, and anxiety: Mini-Mental State Examination (MMSE), Visual Analog Scale (VAS), Beck Depression Inventory (BDI), State-Trait Anxiety Inventory (STAI) and Generalized Anxiety Disorder Screener (GAD).

Fifty-four patients were recruited, but 5 were discarded for not completing evaluations. Forty-nine participants were included in the study and divided into two groups according to the type of CPP they experienced. If the patient suffered from CPP only during her menstrual period, she was classified in the cyclical CPP group (*n *= 21), if the patient presented persistently CPP regardless of the menstrual phase, she was classified in the group of non-cyclical CPP group (*n* = 28). A psychometric evaluation was performed when the patients with cyclical CPP were in the menstrual phase, while the patients with non-cyclical pain reported permanent pain during the menstrual phase. Then, the evaluation was performed in the same phase of the cycle.

### Instruments

The Mini-Mental State Examination (MMSE) is a test used to detect mild cognitive impairment through tests of orientation, memory, attention, calculation, and language. If the score is ≤ 24, probable cognitive impairment is suspected and if it is > 24, the result was "without cognitive impairment" [30].

Wong-Baker FACES® Pain Rating Scale is a visual analog scale (VAS) that self-reported the intensity of CPP. The scale is made up of six faces drawn with ratings from 0 to 10, where 0 is equivalent to the minimum pain and 10 to the maximum pain that have experienced [31].

Beck Depression Inventory (BDI) is a 21-item measure of depression equivalent to the Diagnostic and Statistical Manual of Mental Disorders (DSM) symptoms of depression [32]. Patients chose their responses on a 0–3 Likert-type scale. Scores of BDI can range from 0 to 63 with the following cut- offs: 0–13, minimally depressed; 14–19, mildly depressed, 20–28, moderately depressed, and 29–63, severely depressed [33, 34].

State-Trait Anxiety Inventory (STAI) is used to measure two different dimensions of anxiety: State Anxiety Scale evaluated the current state of anxiety, asking how patients feel “right now”; and Trait Anxiety Scale evaluated relatively stable aspects of “anxiety proneness” [35]. Scores of both scales range from 20 to 80. Scores between 20 and 31 indicated minimal anxiety, 32 to 43 mild anxiety, 44 to 55 moderate anxiety, 56 to 67 severe anxiety, and 68 to 80 maximum anxiety [36].

Generalized Anxiety Disorder Screener (GAD) is a 7-item self-report for screening of Generalized Anxiety Disorder which are rated on a 4-point Likert-type indicating symptom frequency, ranging from 0 (not at all sure) to 3 (nearly every day), yielding a value in the response range from 0 to 21 points. Higher scores indicate higher levels of GAD symptoms [37]. All the instruments have been translated to the local language and validated in the local setting [33, 36, 37].

### Statistical analysis

Demographic parameters and sociomedical conditions were expressed as mean ± SD or N (%), Bonferroni's correction was used to reduce type 1 error. Inferential analysis was performed with a chi-square (nominal variables), Mann–Whitney U (ordinal variables), and Student’s t-test for independent samples (scalar variables) to compare the difference between groups. Relative risk estimation was performed to determine the association between non-cyclical and cyclical CPP with probability of presenting emotional dysregulation. Statistical analyzes were performed with SPSS v.24.0 software (Armonk, New York: IBM Corp). For all statistical analyses, *p* < 0.05 was considered significant.

## Results

### Demographics characteristics

Table 1 shows the sociodemographic characteristics of women with endometriosis with cyclical (*n* = 21) and non-cyclical CPP (*n* = 28). There were no differences in age, years of study, working status and marital status between the two groups. However, results indicate that only 23.8% of women with cyclic pain and 53.5% with non-cyclical pain were married or cohabiting.

**Table 1:** Sociodemographic characteristics of endometriosis women with CPP

**Participants**	**Cyclical pain**	**Non-cyclical pain**	***p*-value**
*n* = 49	*n* = 21	*n* = 28	
**Age**	**Mean (SD)**	**Mean (SD)**	**.80**
	35.2 (6.9)	34.7 (6.47)	
**Marital status**	**N (%)**	**N (%)**	**.36**
Never married	15 (71.4)	10 (35.7)	
Married	3 (14.3)	9 (32.1)	
Divorced	1 (4.8)	3 (10.7)	
Cohabiting	2 (9.5)	6 (21.4)	
**Years of study**	**Mean (SD)**	**Mean (SD)**	**.40**
	14.4 (3.4)	15.1 (3.13)	
**Working status**	**N (%)**	**N (%)**	**.96**
Employee	5 (23.8)	5 (17.9)	
Unemployed	2 (9.5)	4 (14.3)	
Home labor	5 (23.8)	7 (25)	
Commerce	3 (9.5)	4 (14.3)	
Profession	4 (19.4)	6 (21.4)	
Study	1 (4.8)	2 (7.1)	

The parametric t-test was used to detect statistical differences between demographic measures age, years of study. The chi-square test was used to determine differences in marital status, working status between women with cyclical and non-cyclical pain. Bonferroni´s correction was used. *n* = 49

Medical characteristics of patients are described in Table 2. The percentage of nulliparous women is higher in women with non-cyclical CPP (78.6%) than in cyclical CPP women (45.6%). In both cases about 60% of patients report disabling pain for about 10 years and more than 70% of all women described at least another symptom associated with endometriosis. Most patients in both groups have received at least one surgery to manage symptoms including cleaning of endometrial focuses by laparoscopy (conservative surgery), which was the most common surgery in these patients. Additionally, all women reported consumption of some drug for the endometriosis symptoms, mainly non-steroidal anti-inflammatory drugs (NSAIDs). No differences were found in disabling CPP perception, years reporting disabling pain, other presenting symptoms, previous surgery endometriosis, or disruptions, comorbidities between women with cyclical and non-cyclical pain.

**Table 2:** Medical conditions of endometriosis women with CPP

	**Cyclical pain**	**Non-cyclical pain**	***p*-value**
**Parity**^**a**^	**N (%)**	**N (%)**	.024*
Nulliparous	10 (45.6)	22 (78.6)	
≥ 1	11 (52.4)	6 (21.4)	
**Disabling CPP perception**	**N (%)**	**N (%)**	**.61**
Yes	12 (57.1)	18 (64.3)	
No	9 (42.9)	10 (35.7)	
**Years reporting disabling CPP**	**Mean (SD)**	**Mean (SD)**	.51
	9.38 (8.36)	10.8 (8.10)	
**Other symptoms**^**a**^	**N (%)**	**N (%)**	
No other^b^	7 **(**33.3)	7 (25)	.52
Menorrhagia	6 (28.6)	14 (50)	.131
Dyspareunia	7 (33.3)	10 (35.7)	.862
Widespread pain	2 (9.52)	4 (14.3)	.615
Amenorrhea	2 (9.52)	3 (10.7)	.892
Chronic fatigue	1 (4.76)	4 (14.3)	.276
Inflammation	4 (19.0)	1 (3.57)	.077
Rectal tenesmus	0	2 (7.14)	.211
**Infertility**	0	2 (7.10)	..211
Dysuria	1 (4.76)	1 (3.57)	.835
Premenstrual dysphoria	1 (4.76)	0	.243
Subinfertility	1(4.8)	0	.73
**Previous endometriosis surgery**	**N (%)**	**N (%)**	**.84**
0	6 (28.6)	8 (28.6)	
1	9 (42.9)	10 (35.7)	
≥ 2	6 (28.6)	10 (35.7)	
**Surgery for endometriosis**^**a**^	**N (%)**	**N (%)**	
Endometrial focuses	5 (23.8)	9 (32.1)	.52
Oophorectomy	6 (28.6)	5 (17.9)	.37
Hysterectomy	3 (14.3)	5 (17.9)	.74
Colectomy	2 (9.52)	1 (3.57)	.39
**Pharmacotherapy**^**a**^	**N (%)**	**N (%)**	
NSAIDs^c^	15 (71.4)	26 (92.9)	.04
Hormones	7 (33.3)	8 (28.6)	.72
Antispasmodic	1 (4.8)	3 (10.7)	.45
Anxiolytics	2 (9.52)	2 (7.14)	.76
Opioid analgesic	2 (9.52)	0	.09
Cannabis	0	2 (7.14)	.21
**Disruptions**^**a**^	**N (%)**	**N (%)**	
None	9 (42.9)	5 (17.8)	.11
Work/School	5 (23.8)	10 (35.7)	.37
Relationship	6 (28.6)	8 (28.6)	1
Next surgery	5 (23.8)	6 (21.4)	.84
Social	3 (14.3)	5 (17.9)	.74
Desire to be a mother	3 (14.3)	4 (14.3)	1
Family	0	5 (17.9)	.07
Economy	1 (4.76)	1 (3.57)	.83
**Comorbidities**^**a**^	**N (%)**	**N (%)**	
None	10 (47.6)	19 (67.9)	.15
Polycystic ovary	3 (14.3)	3 (10.7)	.71
Hypothyroidism	3 (14.3)	0	.04
Myomatosis	3 (14.3)	0	.04
Adenomyosis	2 (9.52)	1 (3.57)	.39
Overactive bladder	2 (9.52)	0	.09
Obesity	0	1 (3.57)	.38
Anemia	0	1 (3.57)	.38
Heart disease	1 (4.76)	0	.24

The parametric t-test was used to detect statistical differences between years reporting disabling CPP. The chi-square test was used to determine differences disabling CPP, parity, other presenting symptoms, previous surgery for endometriosis, pharmacotherapy, disruptions, and comorbidities between women with cyclical and non-cyclical pain. *n* = 49; *p < 0.05. Bonferroni´s correction was used

^a^Different options can be associated with the same patient

^b^No other symptoms of endometriosis besides CPP

^c^NSAIDs, Non-steroidal anti-inflammatory drugs

To determine differences in global scores on psychometric scales applied between endometriosis patients with cyclical and non-cyclical CPP, a normal distribution of the results was corroborated with the Shapiro wilk test for *n* ≥ 30 and Levene's test showed equality of variances. Then, the global scores of each scale were analyzed using a Student's t test for independent samples. The global scores obtained in depression, anxiety as a trait and state, and generalized anxiety were higher in women with non-cyclical chronic pain than in those with cyclical pain (Table 3). Student's t test for cognitive impairment could not be calculated because the standard deviation of both groups was equal to 0.

**Table 3:** Cognitive impairment, pain perception, and emotional dysregulation global scores in endometriosis women with cyclical and non-cyclical pain

**Type of chronic pelvic pain**	**Cyclical**	**Non-cyclical**	***p*-value**
Cognitive impairment	28.80 (1.28)	28.35 (1.06)	.11
Pain intensity	7.90 (2.79)	8.85 (1.48)	.13
Depression	11.14 (2.42)	17.46 (1.92)*	.04
Trait anxiety	37.42 (3.23)	47 (2.02)*	.01
State anxiety	39.33 (2.52)	47.35 (1.89)*	.02
Generalized anxiety	5.14 (1.08)	8.46 (1.05)*	.03

Table shows the mean ± SD, *n* = 49, *p < 0.05

**Fig. 1:**
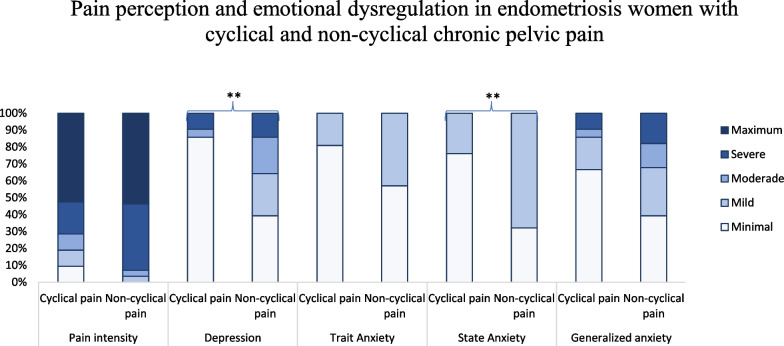
Pain perception and emotional dysregulation in endometriosis women with cyclical and non-cyclical pelvic pain. *n* = 49, **p < 0.01

To determine differences in pain perception and emotional dysregulation between patients with cyclical and non-cyclical CPP according to the clinical classification of each psychometric scales, a Mann–Whitney U test was performed. Most patients with non-cyclical pelvic pain exhibited mild state anxiety (α = 0.007) and depression from mild to severe (α = 0.018) compared to women with cyclical CPP that presented a lower emotional affectation (Fig. 1). No differences were observed in pain intensity, anxiety as a trait or generalized anxiety according to the clinical classification. However, it was found that 70% of endometriosis women with cyclical CPP and more than 90% of the non-cyclical population reported severe to maximum pain; and more than 60% of patients with noncyclic pain presented mild to severe generalized anxiety.

A relative risk estimation was performed to determine the association between non-cyclical or cyclical CPP and the probability of presenting depression or anxiety as risk factors. Results demonstrated a significant relative risk (> 1) in depression (4.5) and state anxiety (2.85) in patients with non-cyclical pain. Relative risk ​​of patients with cyclical chronic pain was not significant (Table 4).

**Table 4:** Relative risks of patients with cyclical and non-cyclical pain

**Type of CPP**	**Depression**	**Trait anxiety**	**State anxiety**	**Generalized anxiety**
**Cyclical**	0.23 (0.07–0.66)	0.44 (0.16–1.18)	0.35 (0.15–0.78)	0.54 (0.28–1.07)
**Non-cyclical**	4.25 (1.43–12.6)^a^	2.25 (0.88–5.99)	2.85 (1.27–6.38)^a^	1.82 (0.92–3.57)

Table shows relative risk scores and SD

^a^Represents a significant relative risk (> 1). *n* = 49

## Discussion

Endometriosis is a long-term, disabling medical condition that affects the quality of life and mental health associated with CPP. Patients with endometriosis may experience CPP in a cyclical manner such as dysmenorrhea or in a noncyclical manner defined as non-menstrual pain. Several reports suggest that chronic experience of pain increases emotional dysregulation [38–40] and that psychiatric disorders are more common among women with endometriosis [41–44], however, differences in emotional dysregulation based on CPP experience in women with endometriosis had not been explored. Therefore, the objective of this study was to determine if there are differences in the levels of emotional dysregulation in patients with cyclical and non-cyclical CPP. This is one of the few studies carried out in Latin America where specialized endometriosis care centers are very limited [29, 45].

High levels of depression and anxiety were found in both groups of patients with CPP, which coincided with previous studies [41–44], however, the present work is the first one in demonstrating higher global scores in depression, anxiety as a trait and state, and generalized anxiety in women with non-cyclical CPP. In addition, more women with endometriosis experiencing non-cyclic CPP suffered from mild to severe depression and mild state anxiety compared to women experiencing cyclical pain. Menorrhagia and persistent pain are two variables that may be associated with greater emotional dysregulation, however, in this study, patients with cyclical pain did not show a difference in the frequency of these symptoms compared to patients with non-cyclical pain [46, 47]. However, it is essential to consider the complexity of the disease and the emotional care of these women to improve their quality of life. Relative risk estimation associated with pain intensity determined a higher probability of developing depression, and state anxiety in patients with non-cyclical pain. In fact, the risk of presenting emotional disturbances is more than doubled in the group of women with noncyclic pain than in those with cyclical pain, which gives us clinically significant and relevant data for the diagnosis and management of these patients [48, 49].

Pain intensity was assessed using the VAS, since it has been shown to be effective for most patients with endometriosis (64%) during the painful experience and indeed, one month after the experience [50]. However, no statistically significant differences were found in intensity of pain between CPP groups. In both cases most patients report severe to maximum pain and perceive it as a disabling pain for about a decade, which could significantly affect their quality-of-life [51]. The relationship between reports of pain and physical pathology is still debated. Authors suggest a complete evaluation of the pain considering location, duration, sensory and affective description, functional status in daily activities [52]; and the hours or sleep disturbances derived from pain [23]. Besides, Api [53] highlights that other symptoms of endometriosis such as painful intercourse or dyspareunia can mediate the intensity of CPP; however, in this study no differences were found in other symptoms of endometriosis between patients with cyclical and non-cyclical pain.

For the management of endometriosis symptoms, all the patients reported drug use, mainly analgesics. Because cognitive impairment is common in patients with chronic pain for excessive use of analgesics including opioids, increased vulnerability to endocrine disrupting chemicals, and age-related cognitive decline [54–56], MMSE test was applied. However, no cognitive impairment was found in women with endometriosis using MMSE. Nevertheless, cognitive impairments were reported by Wassink [57], through EGG and event-related potentials in these patients. It is recommended to explore specific cognitive functions with neuropsychological batteries to improve rehabilitation for future studies [58].

In this study, most patients reported disruptions associated with symptoms of endometriosis, at work, relationships and family. In addition, it was observed that most women with cyclical pain had not been married, and most women with non-cyclical pain do not have children. Low social support and family networks must maintain depressive and anxious states [59, 60], so women with endometriosis may be more vulnerable to living with chronic emotional dysregulation, which is associated with low quality of life [45]. Marital status and number of children are not predictors of emotional well-being in midlife in women, but rather the quality of relationships [51, 61, 62]. Intensity of pain and emotional dysregulation in women with endometriosis can be mediated by psychosocial variables such as emotional suppression, pain catastrophism, personality, and a passive coping style, which can also affect patients’ interactions [63–65].

Different comorbid conditions have been implicated in CPP in endometriosis, such as pelvic floor tenderness, painful bladder syndrome, sexual assault, higher body mass index, current smoking, physical activity, depression, and anxiety [66, 67]. This is the first study that describes differences in emotional dysregulation according to the type of CPP experienced by patients with endometriosis. Therefore, continued research is required to validate these psychosocial factors and determine if any of them is potentially modifiable for improving the quality of life of women with endometriosis.

## Conclusions

Our data suggest that non-cyclical (persistent) CPP is associated with a higher emotional dysregulation than those with cyclical pain women with endometriosis, and that non-cyclical CPP may make patients more vulnerable to developing emotional dysregulation.

## Data Availability

The datasets generated and/or analyzed during the current study are not publicly available due institutional policies but are available from the corresponding author on reasonable request.

## Abbreviations

CPP: Chronic pelvic pain
INPer: National Institute of Perinatology
MMSE: Mini-Mental State Examination
VAS: Visual Analog Scale
BDI: Beck Depression Inventory
DSM: Diagnostic and Statistical Manual of Mental Disorders
STAI: State-Trait Anxiety Inventory
GAD: Screener
NSAIDs: Non-steroidal anti-inflammatory drugs
